# Experimental and Numerical Evaluation of the Mechanical Behavior of Strongly Anisotropic Light-Weight Metallic Fiber Structures under Static and Dynamic Compressive Loading

**DOI:** 10.3390/ma9050398

**Published:** 2016-05-21

**Authors:** Olaf Andersen, Matej Vesenjak, Thomas Fiedler, Ulrike Jehring, Lovre Krstulović-Opara

**Affiliations:** 1 Fraunhofer Institute for Manufacturing Technology and Advanced Materials, Branch Lab Dresden, D-01277 Dresden, Germany; ulrike.jehring@ifam-dd.fraunhofer.de; 2 Faculty of Mechanical Engineering, University of Maribor, Smetanova 17, SI-2000 Maribor, Slovenia; matej.vesenjak@uni-mb.si; 3 Centre for Mass and Thermal Transport in Engineering Materials, School of Engineering, The University of Newcastle, Callaghan, NSW 2308, Australia; Thomas.Fiedler@newcastle.edu.au; 4 Faculty of Electrical Engineering, Mechanical Engineering and Naval Architecture, University of Split, R. Boškovića 32, HR-2100 Split, Croatia; Lovre.Krstulovic-Opara@fesb.hr

**Keywords:** aluminum fiber, fiber structure, orthotropy, sintering, compression, static loading, dynamic loading, energy absorption, numerical simulation

## Abstract

Rigid metallic fiber structures made from a variety of different metals and alloys have been investigated mainly with regard to their functional properties such as heat transfer, pressure drop, or filtration characteristics. With the recent advent of aluminum and magnesium-based fiber structures, the application of such structures in light-weight crash absorbers has become conceivable. The present paper therefore elucidates the mechanical behavior of rigid sintered fiber structures under quasi-static and dynamic loading. Special attention is paid to the strongly anisotropic properties observed for different directions of loading in relation to the main fiber orientation. Basically, the structures show an orthotropic behavior; however, a finite thickness of the fiber slabs results in moderate deviations from a purely orthotropic behavior. The morphology of the tested specimens is examined by computed tomography, and experimental results for different directions of loading as well as different relative densities are presented. Numerical calculations were carried out using real structural data derived from the computed tomography data. Depending on the direction of loading, the fiber structures show a distinctively different deformation behavior both experimentally and numerically. Based on these results, the prevalent modes of deformation are discussed and a first comparison with an established polymer foam and an assessment of the applicability of aluminum fiber structures in crash protection devices is attempted.

## 1. Introduction

In contrast to textile fleeces, felts, and other non-bonded fiber-based structures, the present investigation focuses on the mechanical behavior of rigid fibers structures made from sintered short metallic fibers. By and large, they belong to the realm of metallic foams or so-called cellular metals. Some research has been performed on the determination of mechanical properties of likewise rigid metallic fiber structures. The available literature on such investigations has been summarized by Veyhl *et al.* [1] and goes back as early as 1978 when Ducheyne *et al.* [2] manufactured sintered fiber structures from stainless steel 316L with a relative density of 0.4 and investigated their tensile and compressive properties. In the present paper, the quasi-static test results cited in [1] have been re-evaluated with regard to energy absorption. These results are complemented by new dynamic compression tests performed on a different set of samples which are accompanied by the simulation of the deformation behavior in order to gain more insight into the governing failure mechanisms.

When comparing results of different research groups, the direction of loading with regard to the morphology of the fiber structure and the nature of the inter-fiber bonds has to be taken into account very carefully as they influence results considerably, *i.e.*, the quantification of the fiber structure anisotropy and its influence on the stiffness of the fiber network has been addressed in [3]. This work has been carried out on steel fiber structures where the connection between the fibers was made by a brazing process using copper as the braze material. In contrast, other work on steel fiber structures used vacuum or protective atmosphere sintering (*i.e.*, [4]); therefore, it can be expected that the strength of the inter-fiber bonds should be significantly different in the latter case.

All previous references reveal the same behavior where an increase in relative density results in rising mechanical properties. This is also the case in the present study. The relative density is defined as:

ρ_r_ = m_S_/(V_S_ × ρ), (1)

where ρ_r_ is the relative density, m_S_ the mass of the sample, V_S_ the volume of the sample, and ρ the bulk density of the fiber material.

The aforementioned research was mainly based on experimental quasi-static testing (*i.e.*, uni-axial tensile and compression testing). In contrast, the current research focuses on the energy absorption and the influence of structural anisotropy both under quasi-static and dynamic loading. It was found that the direction of loading with regard to the anisotropy of the fiber structure exerts a significant influence on the mechanical properties. Moreover, the deformation and failure mechanisms are visibly different. Numerical simulation confirmed a high anisotropy of sintered metal fiber structure (SMFS) specimens. In the case of the loading direction parallel to the main fiber orientation they revealed that the deformation is distributed along the metallic fibers (with main local fiber deformation mechanisms: buckling and bending) while, in the case of the loading direction perpendicular to the main fiber orientation, a layer-wise deformation mode (with main local fiber deformation mechanisms: bending and compression in the radial direction) has been observed.

In addition, the energy absorption during dynamic loading was found to be smaller than under quasi-static loading. This could be attributed to the dynamic softening due to a rise in temperature of the samples during testing and early failure of fibers and their sintered bonds due to buckling and local bending. Due to the pronounced orthotropy of the fiber structures, a strong dependence of the deformation behavior on the direction of loading can be observed, *i.e.*, loading parallel to the preferred fiber orientation results in a strong expansion of the samples accompanied by an initially strong rise in stress, whereas loading perpendicular to the main fiber orientation leads to almost no changes in the horizontal projection of the sample shape.

The performance of the aluminum fiber structures in terms of energy absorption was compared to that of a commercially available, closed-cell, high-density polymer foam. At comparable stress and strain levels, the weight specific performance of an aluminum fiber sample with a relative density of 0.16 reached 12% and the volume specific performance 50% of the values of the polymer foam. This is better than expected and could be further optimized by using materials with better strength-to-weight ratios such as high-strength aluminum alloys, as well as titanium or steel fibers.

## 2. Results

### 2.1. Quasi-Static Test Results

**Figure 1:**
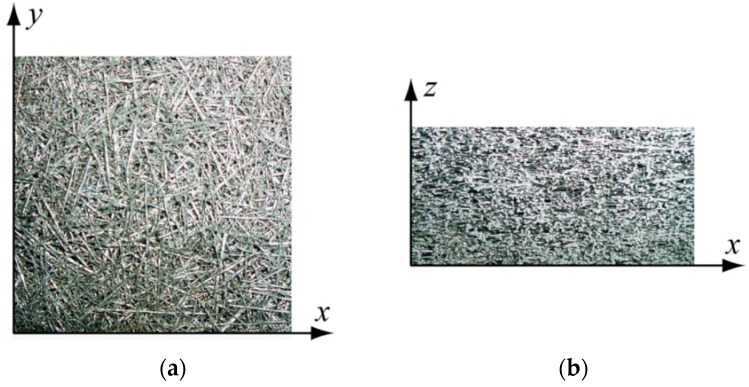
Orthotropy of sintered short fiber structures: (**a**) view perpendicular to preferred fiber orientation; (**b**) view parallel to preferred fiber orientation. Accordingly, the *z*-direction corresponds to the direction of loading perpendicular to the preferred fiber orientation while loading along the *x*-*y* plane corresponds to loading parallel to the preferred fiber orientation [1].

For quasi-static testing, a procedure as described in Section 4.2 was employed. Two different directions of loading were employed as illustrated in Figure 1.

**Figure 2:**
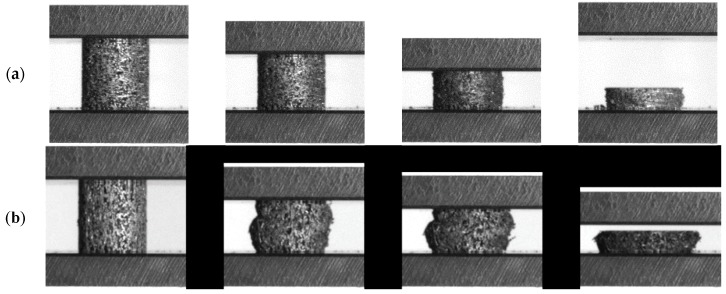
Photographs of the quasi-static sample deformation in dependence of the direction of loading: (**a**) Loading perpendicular to main fiber orientation (relative density of the sample: 0.27); and (**b**) loading parallel to main fiber orientation (relative density of the sample: 0.22).

Figure 2 shows photographs of the progression of the compression in dependence of the direction of loading. As can be seen, the deformation behavior is strongly dependent on the direction of loading. Former compression tests performed on other sintered short fiber structures showed basically the same dependence; therefore, it can be concluded that this behavior is caused by the specific morphology of these structures.

**Figure 3:**
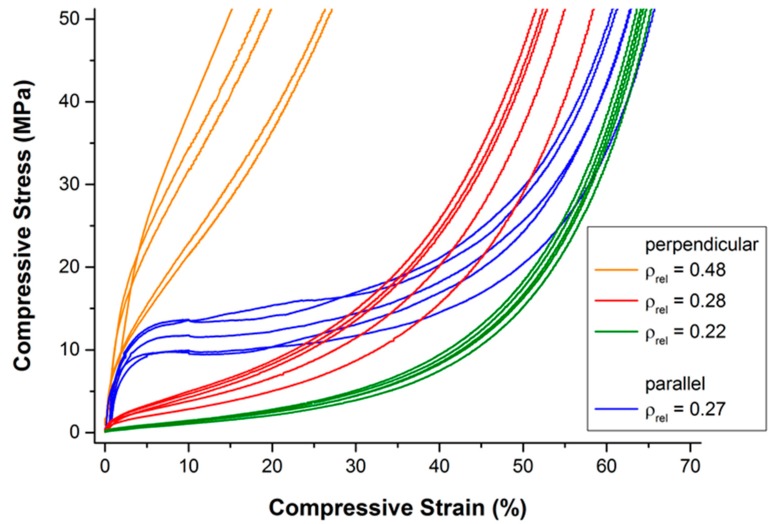
Compressive quasi-static stress-strain curves in dependence of relative density and the direction of loading.

The resulting compressive stress-strain curves are given in Figure 3. With the exception of the sample with the highest relative density of 0.48, the observed stress-strain curves follow the typical pattern of open- and closed-cell cellular metals under compressive loading: an initial strong rise in stress (quasi-elastic region) is followed by a comparably moderate rise in stress (plateau region; characterized mainly by bending and occasional failure of the struts or cell walls) and a third region with another strong rise in stress (densification region; formerly separate struts or cell walls start touching each other, heavy deformation and failure of single struts or cell walls occur).

A strong influence of relative density and direction of loading can be observed. Note that the set of curves of samples of almost the same relative density (0.27 and 0.28, respectively) but different direction of loading are intersecting at a relative deformation of approximately 25% to 50%. In other words, loading parallel to the main fiber orientation results in a more “typical” compression behavior: a pronounced quasi-elastic rise in the beginning, a comparably long and flat plateau region, and, finally, a strong rise in stress due to the onset of densification. Deviations within one set of samples can be most likely attributed to variations of density of the individual samples (see also Section 4.1).

### 2.2. Dynamic Test Results

**Figure 4:**
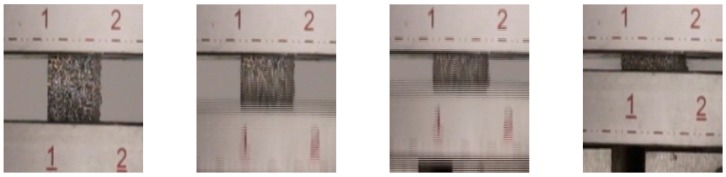
Progression of dynamic loading. Testing direction: perpendicular to main fiber orientation (time interval between consecutive pictures ≈ 0.01 s). Relative density of the sample: 0.30.

**Figure 5:**
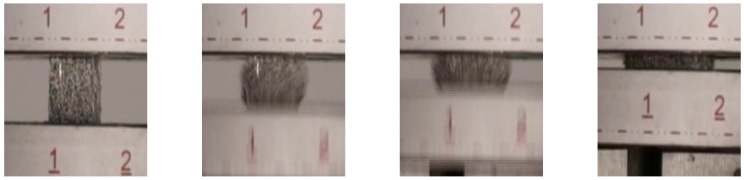
Progression of dynamic loading. Testing direction: parallel to main fiber orientation (time interval between consecutive pictures ≈ 0.01 s). Relative density of the sample: 0.16.

For dynamic testing, a procedure as described in Section 4.2 was employed. Figure 4 and Figure 5 show photographs of the progression of the compression in dependence of the direction of loading. As has already been observed during quasi-static testing, the deformation behavior clearly depends on the direction of loading.

**Figure 6:**
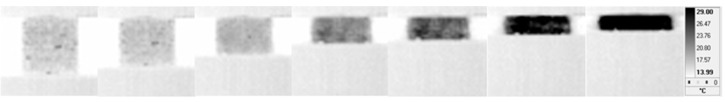
IR images for loading perpendicular to main fiber orientation (relative density: 0.3). Time interval between consecutive pictures ≈ 0.005 s.

**Figure 7:**
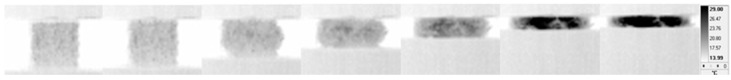
IR images for loading parallel to main fiber orientation (relative density: 0.3). Time interval between consecutive pictures ≈ 0.005 s.

**Figure 8:**
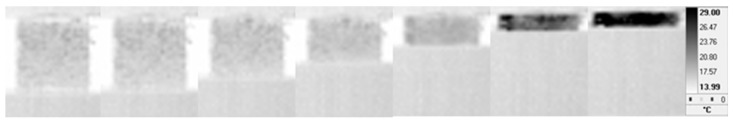
IR images for loading perpendicular to main fiber orientation (relative density: 0.16). Time interval between consecutive pictures ≈ 0.005 s.

**Figure 9:**
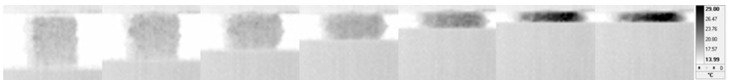
IR images for loading parallel to main fiber orientation (relative density: 0.16). Time interval between consecutive pictures ≈ 0.005 s.

Figure 6, Figure 7, Figure 8 and Figure 9 depict the infrared (IR) thermography recordings. In Figure 6, the main fiber orientation of the specimen with relative density of 0.3 is perpendicular to the loading direction. This case is characterized by localization of surface plastification in six zones close to the loading plates. The same behavior occurs for the case of 0.16 relative density (Figure 8) but for this density plastification localization is clearly visible in two zones. Less proclaimed plastification could be linked with lower heat generation and lower dissipation of energy due to lower relative density. The deformation process along this orientation is showing a deformation behavior typical for open-cell cellular specimens, *i.e.*, no barreling, but a layer-wise collapse mechanism, leading to lower yielding and lower plateau stress.

Figure 7 and Figure 9 show the deformation where the main fiber orientation is parallel to the loading direction for specimens with relative density of 0.3 and 0.16, respectively. In both cases buckling and significant barreling occur. This effect is more proclaimed in the case of higher relative density. Behavior of such specimens is more similar to a bulk material. They experience higher yield stress, after which the stress slightly decreases due to fiber buckling and finally increases again towards the densification.

**Figure 10:**
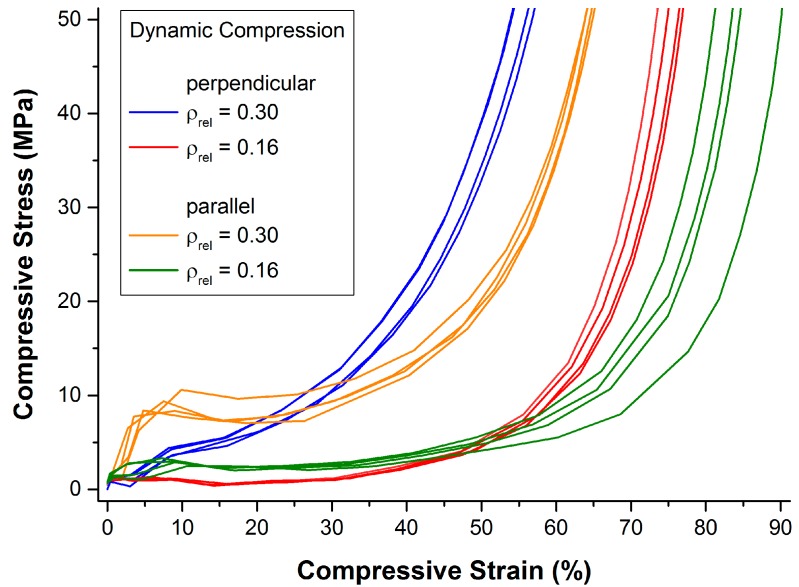
Compressive dynamic stress-strain curves in dependence of the relative density and the direction of loading.

The resulting compressive stress-strain curves are given in Figure 10. Generally, as is also the case in quasi-static testing, the observed stress-strain curves follow the typical pattern of open- and closed-cell cellular metals under compressive loading: an initially strong rise in stress (quasi-elastic region) is followed by a comparably moderate rise in stress (plateau region; characterized mainly by bending and occasional failure of the struts or cell walls), and a third region with another strong rise in stress (densification region; formerly separate struts or cell walls start touching each other, heavy deformation, and failure of single struts or cell walls occurs).

A strong influence of the relative density and the direction of loading on the measured stress can be observed. In particular, note that the set of curves of samples of the same relative density but different direction of loading are intersecting at a relative deformation of approximately 20% and 55%, respectively. In other words, loading parallel to the main fiber orientation results in a more “typical” compression behavior: a pronounced quasi-elastic rise in the beginning, a comparably long and flat plateau region, and, finally, a strong rise in stress due to the onset of densification. In that regard, there seems to be no difference between quasi-static and dynamic testing conditions.

Deviations within one set of samples can be most likely attributed to variations of density of the individual samples. In general, deviations within one set of samples are smaller than in quasi-static testing which corresponds well to the smaller relative deviations in density compared to the quasi-static test samples (see also Section 4.2).

## 3. Discussion

### 3.1. Mechanical Behavior

Two distinct deformation modes can be observed depending on the direction of loading relative to the main fiber orientation. At the same sample relative density level, loading parallel to the main fiber orientation results in an initially much stiffer behavior than loading perpendicular to the main fiber orientation. However, under both quasi-static and dynamic loading, we observe that at higher deformations (typically between 20% and 55% strain depending on testing direction and density of the samples) the initially stiffer parallel testing direction becomes softer than the perpendicular testing direction. The different sample deformation behavior as shown earlier in Figure 2 indicates that this may be attributed to buckling of the fibers, breaking of fibers, and disruption of sinter bonds between the fibers. Parallel loading obviously results in an expansion of the sample cross-section, whereas perpendicular loading does hardly change the sample cross-section even at high levels of compression. It is assumed that deformation perpendicular to the main fiber orientation is for the most part resembling a folding-like action (much like a pantograph) until densification starts. Although a more thorough experimental investigation of this hypothesis has not been carried out, the results of the numerical calculations support this conclusion.

**Figure 11:**
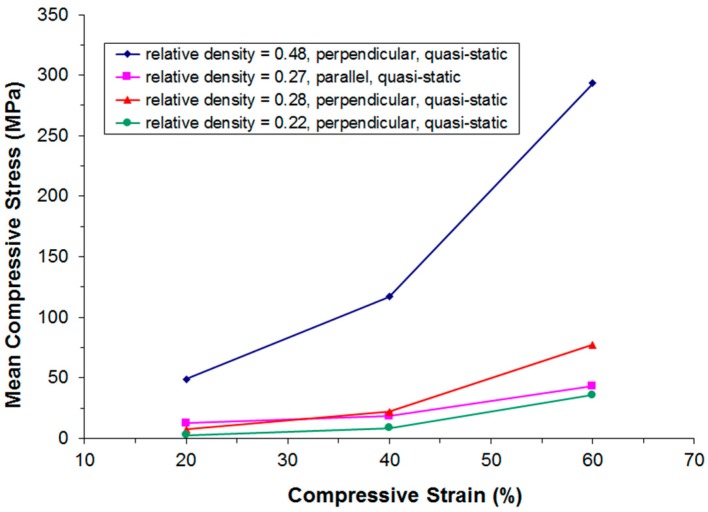
Compressive mean stress values at 20%, 40%, and 60% strain derived from quasi-static stress-strain curves in dependence of relative density and the direction of loading.

**Figure 12:**
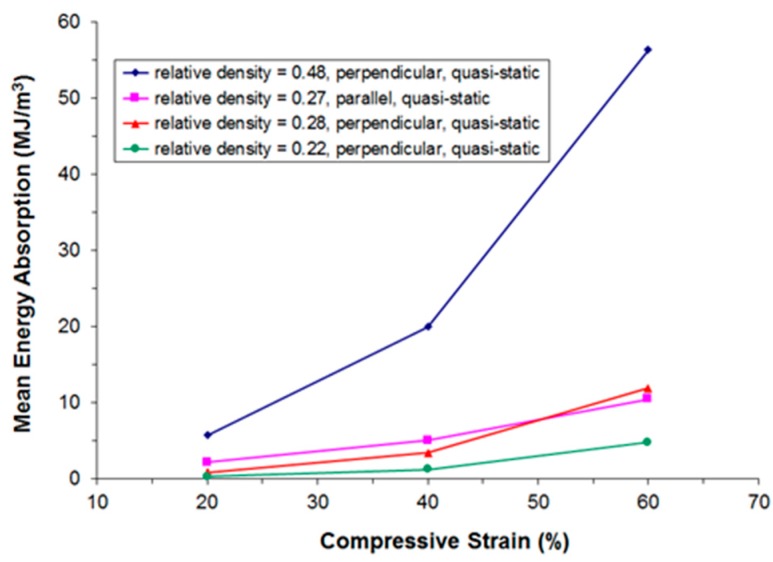
Compressive mean specific energy absorption values at 20%, 40%, and 60% strain derived from quasi-static stress-strain curves in dependence of relative density and the direction of loading.

**Figure 13:**
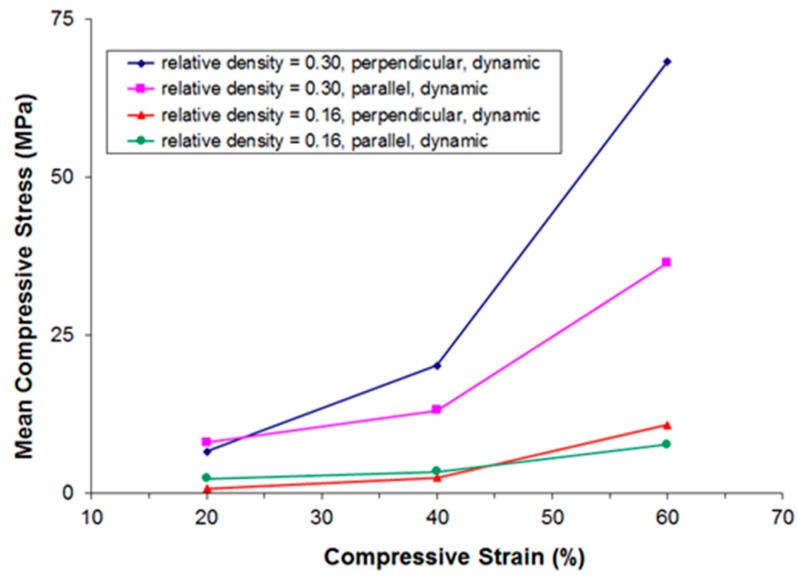
Compressive mean stress values at 20%, 40%, and 60% strain derived from dynamic stress-strain curves in dependence of relative density and the direction of loading.

**Figure 14:**
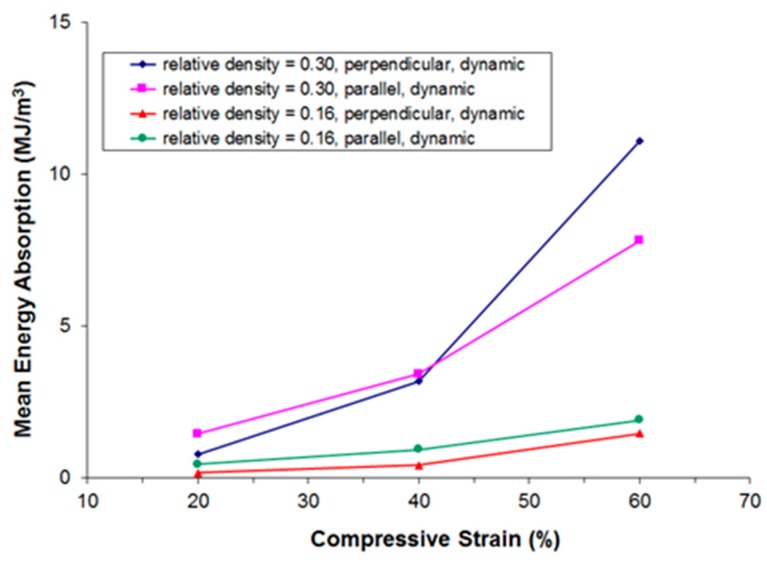
Compressive mean specific energy absorption values at 20%, 40%, and 60% strain derived from dynamic stress-strain curves in dependence of relative density and the direction of loading.

Figure 11, Figure 12, Figure 13 and Figure 14 show the compressive mean stress values at 20%, 40%, and 60% strain and the corresponding mean specific energy absorption values both for quasi-static and dynamic testing. The specific energy absorption value is calculated by integrating force over displacement up to a given strain value. This result is then divided by the volume of the sample prior to compression, thereby providing a basis for comparison of the performance of different materials. DIN 50134 (testing of metallic materials—compression test of metallic cellular materials) suggests to determine energy absorption values at 20%, 40%, and 60% deformation, which is the reason for taking the specific energy absorption values at these strain levels. Otherwise, there is no specific physical reason for taking these values.

Again, we observe that for samples of comparable density, both under quasi-static and dynamic loading the initially stiffer parallel testing direction becomes softer than the perpendicular testing direction at larger deformations.

**Figure 15:**
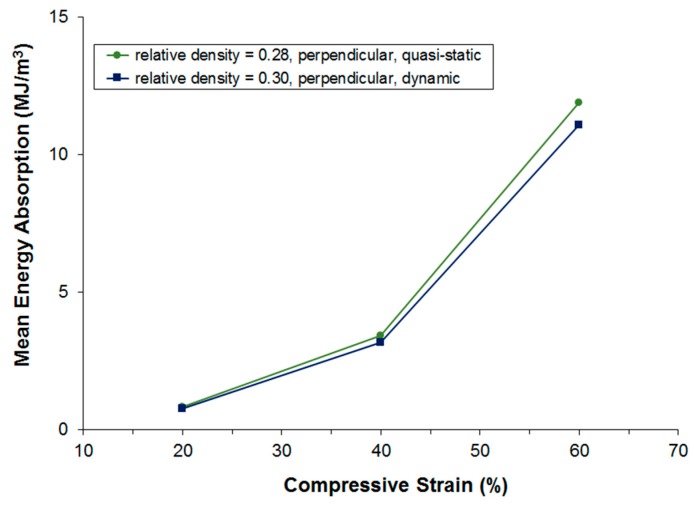
Comparison of static and dynamic energy absorption at different strain levels. Loading perpendicular to main fiber orientation.

**Figure 16:**
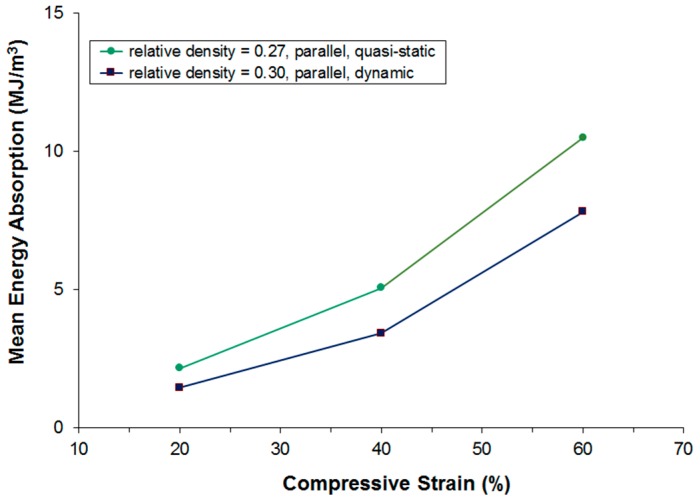
Comparison of static and dynamic energy absorption. Loading parallel to main fiber orientation.

Figure 15 and Figure 16 show a comparison of the energy absorption at different strain levels under quasi-static and dynamic loading. Unfortunately, the mean density of the samples compared is not exactly the same; however, it is still clearly visible that energy absorption is lower during dynamic testing even though the dynamic testing samples show a higher density. The real difference in energy absorption should, thus, be even higher and it can, therefore, be concluded that under the given testing conditions, the dynamic energy absorption is at least 10% lower in the perpendicular testing direction and at least 35% lower in the parallel testing direction.

In [5], several mechanisms are discussed which lead to either softening or strengthening during dynamic compression of metal foams. However, strengthening effects like the micro-inertial or shockwave propagation effects can be expected only at much higher testing speeds than those applied here. Additionally, strengthening due to gas compression inside pores can be ruled out as the fiber structures feature a completely open and interconnected porosity.

On the other hand, plastic deformation of metals is occurring through a number of thermally activated processes such as dislocation glide. As was already shown earlier, the samples undergo measurable heating-up during dynamic testing. Average temperature rises of up to 20 K were measured although the loading velocity for the tested specimens was moderate (284 mm/s). However, as the spatial resolution of the IR thermography is low and heat dissipates quickly along the fibers, the local temperature rise at the actual sites of plastic deformation is assumed to be much higher. Therefore, the lower energy consumption during dynamic testing is attributed to dynamic softening of the samples due to the rise in temperature at the very sites of plastic deformation.

It is conceivable that the pronounced dependence of the compression behavior on the testing direction might be due to a prevalence of fiber buckling and rupture of bonds in the direction parallel to the main fiber orientation. This hypothesis is strongly supported by the numerical calculations.

In addition to the general difference in the deformation behavior in dependence of the testing direction, an influence of the different fiber diameters used for the dynamic and the quasi-static test samples cannot be ruled out. The dynamic test samples with a relative density of 0.3 were made from thicker fibers which thus boast a lower number of inter-fiber bonds. On the other hand, for a fiber with a cylindrical cross section the resistance to buckling scales with the fourth power of the fiber diameter. As the melt-extracted fibers generally show a more flattened-out cross-section, the limiting case of a rectangular cross section may be assumed where the second moment of inertia scales with the third power of the lateral length. Hence, without a very detailed analysis of the average length between the fiber contacts, it is not possible to determine whether the change in distance between the fiber bonds or the increase in buckling strength due to the larger fiber diameter is the dominating effect.

**Figure 17:**
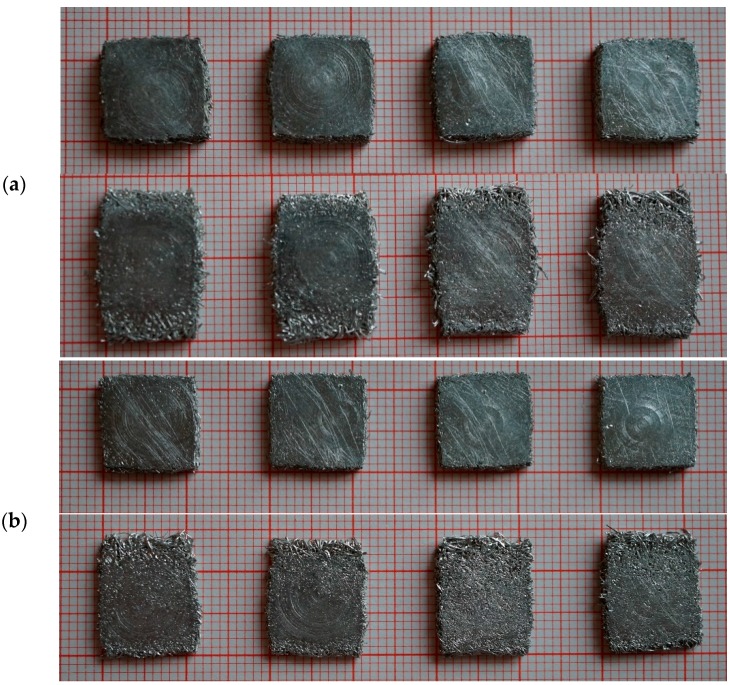
Photographs of sample shapes after dynamic testing: (**a**) Relative sample density 0.3. Upper row: loading perpendicular to main fiber orientation. Lower row: loading parallel to main fiber orientation; and (**b**) relative sample density 0.16. Upper row: loading perpendicular to main fiber orientation. Lower row: loading parallel to main fiber orientation.

Due to the fact that the dynamic testing samples were prepared from a plate-like parent structure as described in Section 4.1, the sample faces constituting the upper and lower sides of the parent structure exhibit slightly different properties as compared to the machined sides. This results in an additional anisotropy of the deformation behavior. The former upper and lower faces of the parent structure consist of a layer of fibers that is completely aligned in one plane; hence, they act much as a sandwich face sheet and outward buckling of this layer occurs, resulting in a pronounced rectangular shape of the compressed samples as shown in Figure 17. In contrast, loading perpendicular to the main fiber orientation results in a quadratic shape of the densified samples as these samples show no additional anisotropy. The higher the density of the sample, the more pronounced this behavior is.

### 3.2. Comparison with High-Density Polymer Foam

Due to their low deformation stress levels, aluminum fiber structures may be compared to polymer foams in terms of specific energy absorption at a given stress level. Here, a commercially available closed-cell polymethacrylimide (PMI) polymer foam (tradename ROHACELL^®^, Evonik Resource Efficiency GmbH, Essen, Germany) shows comparable compressive deformation stress levels and constitutes a relevant benchmark for lightweight crash absorption applications. Chu *et al.* [6] conducted a study where they investigated the crash behavior of ROHACELL^®^ samples in a drop tower. The main parameters of these tests were as follows:

- Sample dia. 80 mm, length 75 mm
- Foam density 110 kg/m^3^
- Drop tower test speed 7 m/s, punch weight 40 kg

**Figure 18:**
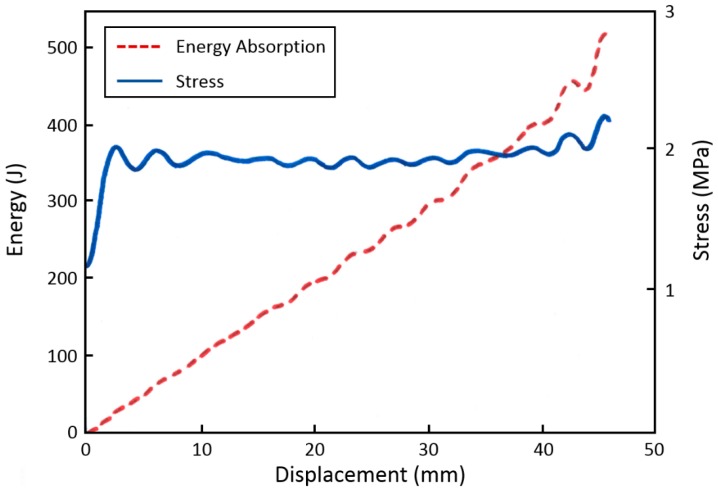
Deformation and energy absorption behavior of ROHACELL^®^ in dynamic compression testing, redrawn from [6]. Sample size was dia. 80 mm, length 75 mm.

From the measured data, a specific energy absorption of 1.23 MJ/m^3^ was calculated (there is an obvious error in the report where it reads 12.3 kJ/m^3^), which was about 25% less than in the quasi-static tests and slightly lower than the data provided by the foam manufacturer. Figure 18 shows the deformation behavior and energy absorption of ROHACELL^®^ 110IG. The foam shows an almost constant stress level (plateau stress) of approx. 2 MPa up to 60% strain. It is, therefore, possible to utilize the foam within this deformation range. The weight specific energy absorption of the tested ROHACELL^®^ specimens at 60% strain EA60 results to 11.2 kJ/kg. The value for EA60 given by the manufacturer is 16.5 kJ/kg; however, the specific test conditions used by the manufacturer are not known.

A direct comparison of the specific energy absorption should be made only at comparable total deformation and stress levels. The tested aluminum fiber structures show a considerable rise in stress after 40% deformation. Therefore, the specific energy absorption at 40% strain (EA40) values of the foam and a suitable fiber structure (sample no. 200743_0043, dynamic testing perpendicular to main fiber orientation) were compared. The EA40 values of the ROHACELL^®^ foam can be taken from Figure 18. The resulting values are a volume specific EA40 of 0.8 MJ/m^3^ and a weight specific EA40 of 7.2 kJ/kg. In comparison, the data of the fiber sample were as follows:

- Fiber material AlCu5, relative sample density 0.16, absolute sample density 480 kg/m^3^
- Calculated volume specific energy absorption up to 40% deformation amounts to 0.41 MJ/m^3^ at a maximum stress of 2.26 MPa. This is reasonably close to the plateau stress value of the polymer foam

The weight specific energy absorption of the aluminum fiber sample amounts to 0.86 kJ/kg which is about 12% of that of ROHACELL^®^ 110IG polymer foam at 40% strain. In terms of volume specific performance, the tested fiber structure is much denser than the polymer foam and, thus, reaches about 50% of the volume specific performance of the polymer foam.

### 3.3. Numerical Results

**Figure 19:**
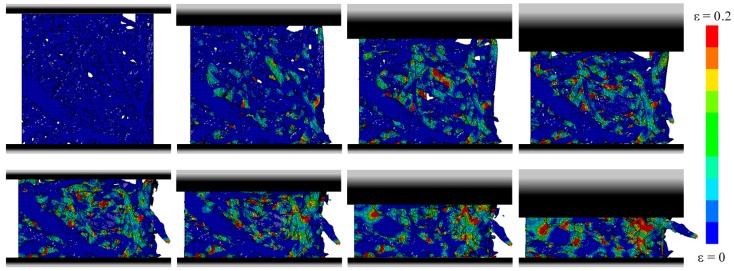
Numerical simulation of the compressive behavior of SMFS subjected to dynamic loading direction parallel to the main fiber orientation (strain rate 30/s, strain increment Δε ≈ 0.1).

**Figure 20:**
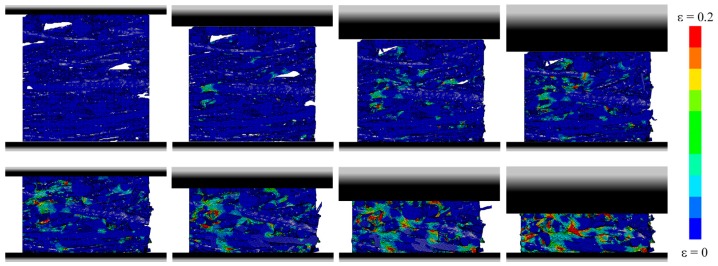
Numerical simulation of the compressive behavior of SMFS subjected to dynamic loading direction perpendicular to the main fiber orientation (strain rate 30/s, strain increment Δε ≈ 0.1).

The dynamic behavior of SMFS specimens was further studied by numerical simulation. The deformation of SMFS subjected to compressive loading parallel and perpendicular to the main fiber orientation is shown in Figure 19 and Figure 20, respectively. In good agreement with the experimental findings, it is observed that the deformation mechanism is distinctively different when changing the loading direction. In case of the loading direction parallel to the main fiber orientation (Figure 19) it can be noted that the deformation (stress) is distributed along the metallic fibers. Consequently, the strain is more evenly dispersed through the specimen’s height. In case of the loading direction perpendicular to the main fiber orientation (Figure 20), a layer-wise deformation mechanism can be observed, as the fibers are stacked onto each other. This results in the weakest horizontal cross-section to strain (stress) concentrations, leading to a layer-wise collapse mechanism, which is repeated until complete densification of the specimen.

**Figure 21:**
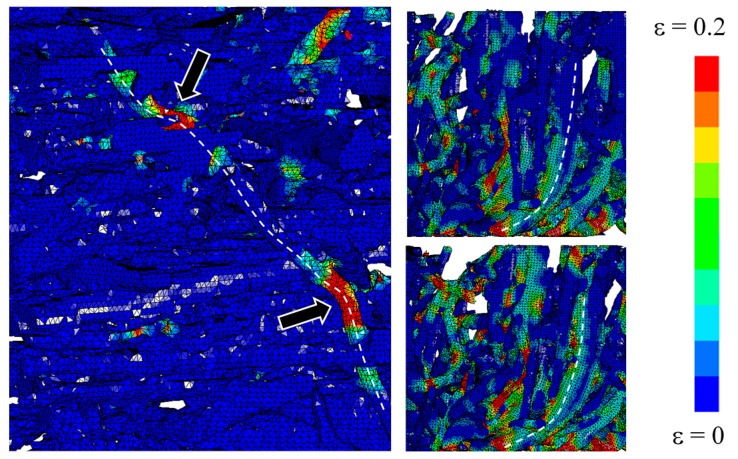
Detailed deformation mechanism in SMFS subjected to loading direction parallel to the main fiber orientation. Arrows indicate buckling and bending of single fibers.

**Figure 22:**
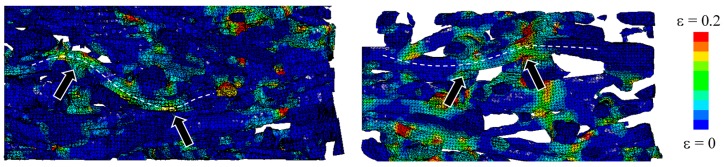
Detailed deformation mechanism in SMFS subjected to loading direction perpendicular to the main fiber orientation. Arrows indicate the local bending of fibers.

The advantage of computational simulation in comparison to experimental testing is the possibility to observe also the specimen’s interior local deformation mechanics in detail. Figure 21 shows the local deformation mechanism of specimens loaded parallel to the main fiber orientation. The most commonly observed deformation mechanisms are buckling and bending. In case of the perpendicular loading direction, local bending of fibers (Figure 22) prevails in combination with compression of fibers in radial direction.

**Figure 23:**
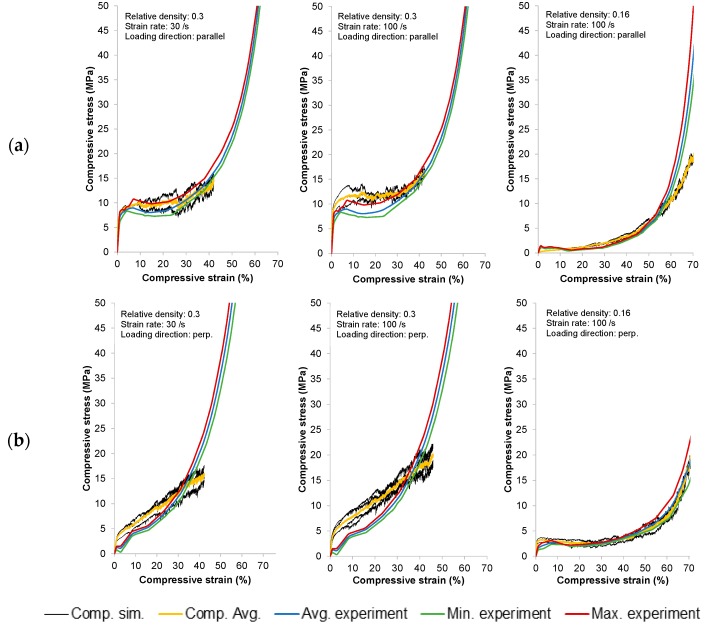
Comparison of numerical and experimental compressive response of SMFS subjected to loading parallel and perpendicular to the main fiber orientation: (**a**) loading parallel to main fiber orientation; and (**b**) loading perpendicular to main fiber orientation.

The comparison between the experimental and numerical response of SMFS with different relative densities (0.3 and 0.16), loading velocities (30/s and 100/s), and directions are shown in the diagrams in Figure 23. In case of the SMSF with higher relative density (0.3), the simulations did not reach the macroscopic strain of 0.7 (as it was the case for specimens with lower relative density) due to massive deformation of the finite elements and their distortion at higher macroscopic deformations. However, excellent agreement can been observed when comparing the result of loading parallel to the main fiber orientation. The start of the plateau region as well as the densification zone can be precisely captured by numerical simulations.

Additionally, a positive strain rate sensitivity of SMFS specimens has been observed. This is in contradiction to the experimental results and can be attributed to the omission of thermal effects in the computational simulations, while the same fiber geometry has been used at different strain rates which was not possible in the case of experimental testing.

In case of the testing direction perpendicular to the main fiber orientation, the numerical response indicates higher stresses in comparison to the experimental measurements for SMFS specimens with higher relative density. The yield stress and plateau stress are higher with respect to the experimental values. The difference in the response might be attributed to the initial compaction of fiber layers (softer in manufactured specimens) in the first loading steps and the connection between single fibers (in simulations, if two or more fibers are in the undeformed SMFS specimen in contact their interfaces are considered as merged). Also, a distinctive transition between the plateau and densification regions could not be captured exactly, which can be mainly attributed to the failure mechanism applied in the numerical models. In case of the perpendicular loading of SMFS specimen with lower relative density again a good comparison through complete macroscopic strain range can be noted.

**Figure 24:**
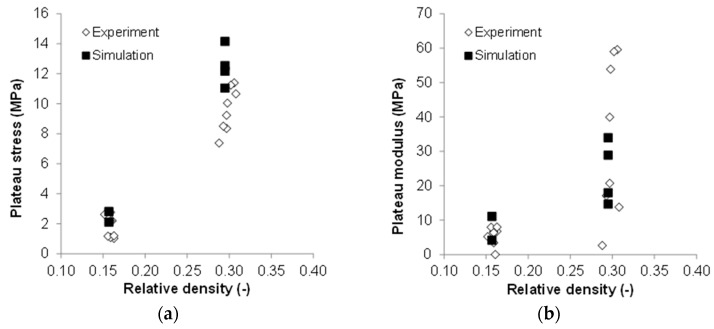
Comparison of experimental and numerical results at a strain rate of 30/s: (**a**) Comparison of experimental and numerical values of the plateau stress in dependence of relative density of the specimens; (**b**) comparison of experimental and numerical values of the plateau modulus in dependence of relative density of the specimens.

The experimental and numerical values of plateau stress and plateau modulus for tested SMFS specimens (both loading directions) are presented in Figure 24. For both mechanical parameters a higher scattering can be observed for SMFS specimens with higher relative density, while in the case of the plateau modulus the numerical results slightly overshoot the experimental values, an excellent agreement can be observed in the case of the plateau modulus.

## 4. Materials and Methods

### 4.1. Sample Manufacturing

In order to produce aluminum fiber structures, a liquid phase sintering route has been developed requiring aluminum alloy fibers with appropriate composition and clean surface. This can be accomplished by utilizing the crucible melt extraction (CME) process by which it is possible to manufacture short fibers from almost any fusible material. To this end, a rotating wheel with a notched surface is placed over a melt pool. The rotating extraction device is water cooled and, thus, generates a high solidification rate. As a result, homogenous distribution of the alloying elements, small grain sizes, reduced segregation, and extended solubility, as well as the formation of metastable phases, can be achieved. The melt extracted fibers typically show a sickle or kidney shaped cross-section. Fraunhofer IFAM Dresden has improved the crucible melt extraction process to produce fibers of a mean equivalent diameter from 50 to 250 µm. The mean fiber length is usually in the range of 3 to 25 mm. The manufacturing process is described in greater detail in [7]. The main properties of the fibers used for the production of the test samples are given in Table 1.

Note that the number of fibers contained in a sample of a fixed volume increases linearly with increasing relative density of the sample, holding the fiber diameter constant. This implies that, at the same time, the number of contact points has to increase non-linearly due to the simple fact that the number of contact points approaches infinity with the sample approaching full density or a relative density of 1. This is in contrast to the explanations given in [1] concerning the number of fibers and contact points contained in samples of different densities. Some confusion might arise at this point as there is a considerable amount of literature available that deals with the average contact number of rod-like particles in a packed bed configuration with no external pressure applied. A good summary of numerical approaches to this problem is given in [8]. For instance, simulation of rod packings via molecular dynamics yields average contact numbers of approximately 10 resulting in final jamming of the individual particles and a stable packing. This is in good agreement with other approaches. However, during fiber structure manufacturing, the initially loose packing is strongly compressed during the sintering step, resulting in a considerable rise of the number of contact points as compared to an uncompressed packing.

**Figure 25:**
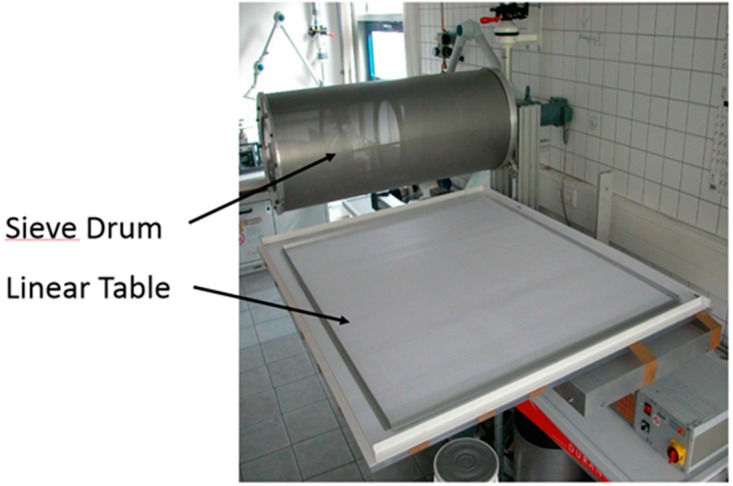
Rotating sieve drum machine used for the manufacturing of the fiber deposits.

The sintered metallic fiber structures reveal a strong orthotropy due to the fiber laying technique. Figure 25 shows the rotating sieve drum machine which is used for the manufacturing of the loose fiber deposits prior to sintering. The drum is filled with fibers which fall onto the linear table moving back and forth underneath the sieve drum. This way, the fibers are preferentially oriented along the plane of the linear table, resulting in an orthotropic morphology (see also [1] for further explanations).

Sintering of the aluminum fibers was carried out in nitrogen or vacuum atmosphere at temperatures slightly above the solidus. For this study, a composition of 95 wt % Al and 5 wt % Cu was used. The amount of liquid phase during sintering is typically around 20 vol %. By applying a weight on top of the fiber deposit during sintering, the porosity of the fiber structures can be set to anywhere between approximately 50% and 90% and is completely interconnected. The pore size usually lies between 10 and 250 µm, depending on the porosity and the fiber diameter.

**Figure 26:**
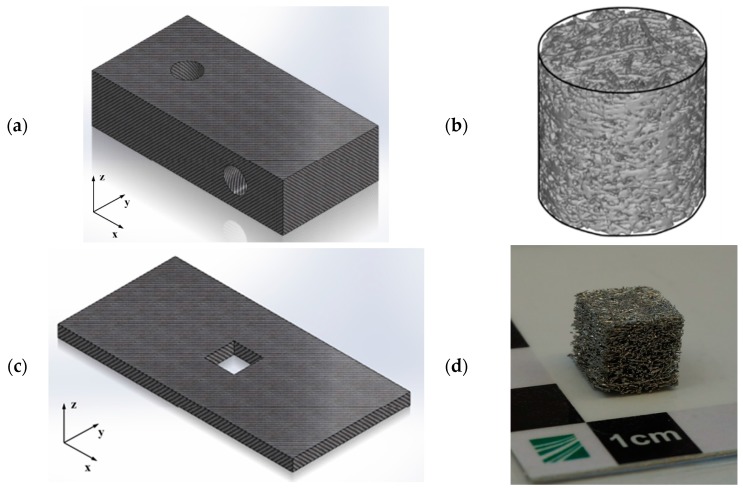
Orientation of static and dynamic test samples within the parent fiber structure: (**a**) brick-like parent structure used for cylindrical samples for quasi-static testing; (**b**) reconstructed CT image of cylindrical sample for quasi-static testing [1]; (**c**) plate-like parent structure for cube-shaped samples for dynamic testing; and (**d**) photograph of cube-shaped sample for dynamic testing. The *z*-direction corresponds to the testing direction perpendicular to the main fiber orientation.

For quasi-static testing, cylindrical samples (diameter 10 mm, length 10 mm) were cut out from a larger block of sintered aluminum fibers by wire electro discharge machining. For dynamic testing, cubes with dimensions 10 × 10 × 10 mm^3^ were prepared from sintered fiber structures (“plates”) of thickness 10 mm. Figure 26 shows the way in which the samples for the quasi-static and dynamic tests were cut out from their respective sintered parent structures. In both cases, the *z*-direction corresponds to the testing direction perpendicular to the main fiber orientation, which is the *x*-*y* plane corresponds to the plane of the linear table during fiber deposition.

Due to the large height of the blocks used as parent structures for the cylindrical samples, a density gradient from top to bottom of the blocks was observed, resulting in a larger scatter of individual sample densities as compared to the dynamic testing samples. The maximum deviation from the mean value within one set of quasi-static test samples reached −7.8% and +6.7%, respectively. In contrast, the maximum deviation from the mean value within one set of dynamic test samples reached only −3.4% and +3.8%, respectively.

### 4.2. Quasi-Static and Dynamic Test Procedure

For quasi-static testing, a procedure in accordance with DIN 50134 was adopted. Testing was carried out at Fraunhofer IFAM, Dresden, Germany, on a Hegewald & Peschke Inspekt table (Hegewald & Peschke Mess- und Prüftechnik GmbH, Nossen, Germany) with a 100 kN load cell. The strain rates were 0.1%/s (corresponding to 0.01 mm/s) between 0% and 10% strain and 1%/s (corresponding to 0.1 mm/s) at higher strain. Compressive loading was achieved by moving the upper plate which is attached to the machine crosshead downwards towards the fixed pressure plate. The finite stiffness of the testing machine was compensated by measuring displacement directly between the pressure plates using an external extensometer.

The dynamic compressive experimental testing was performed at the University of Split, Faculty of Electrical Engineering, Mechanical Engineering and Naval Architecture, Croatia, using the servo-hydraulic testing machine INSTRON 8801 (Instron, Norwood, MA, USA) according to the standard ISO 13314:2011 [9,10]. The number of cubic (10 × 10 × 10 mm^3^) SMFS specimens, their mass, relative density, and testing conditions are given in Table 2.

The loading velocity was set to 284 mm/s which resulted in a macroscopic compressive strain rate of approx. 30/s. The testing machine support plates were lubricated with the graphite-based silicone grease to minimize the friction between the specimens and the support plates. During the tests, the force and cross-head displacement have been recorded to evaluate the compressive mechanical properties. The measured load-displacement data during testing were converted using the initial specimen’s dimensions to compressive stress-strain values.

The experiments were also captured with a Full HD video camera SONY HDR-SR8E (Sony Corporation, Tokyo, Japan), supporting recording at 100 frames per second, and an infrared (IR) thermal camera. The IR thermography allows one to observe and follow the deformation mechanics [11,12]. In [13] it was confirmed that the temperature distribution based on thermal images is equivalent to the strain field on the specimen’s surface. This technique has already been proven to be a reliable tool to analyze the plastification zones, crack propagation, and failure for different types of porous materials, e.g., aluminum foam [14], foam-filled aluminum tubes [15,16], advanced pore morphology (APM) foam elements [17], expanded perlite/aluminum syntactic foam [18], aluminum foam derived from infiltration casting of salt dough [10], and unidirectional porous (UniPore) copper [19].

### 4.3. Numerical Methods

The highly complex geometry of SMFS specimens has been captured and reconstructed based on the micro-computed tomography (μCT) which is already a well-established method for preparation of computational models of various porous materials, *i.e.*, Alporas^®^ [20], M-Pore^®^ [21], Advanced Pore Morphology (APM) foam (Fraunhofer Institute for Manufacturing Technology and Advanced Materials IFAM, Bremen, Germany) [22], expanded perlite/aluminum syntactic foam [18], and aluminum foam derived from infiltration casting of salt dough [10]. The μCT imaging of SMFS samples provided grey level image stacks with a voxel length of 17 μm [1]. After the segmentation, the geometry data was converted into a closed triangulated STL surface mesh which bounds the solid volume of the structure. In the final step, the spatial discretization (meshing with solid finite elements) was performed, where the surface meshes are transformed into volume meshes using the automated meshing software Sharc Harpoon (Sharc Ltd., Manchester, UK). To achieve realistic wall-clock times the size of the computational models were truncated to a cube with the edge length of approx. 2.5 mm. For each relative density (0.3 and 0.16) up to three models have been built.

Geometrical and mechanical convergence of the numerical model requires a sufficient number of finite elements to accurately represent the complex geometry of analyzed structure. To ensure both geometrical and numerical convergence, preliminary parametric sensitivity studies have been performed. It has been observed that ~1.4 million fully integrated tetrahedron finite elements allow to accurately describe the response of the considered SMFS model [1,23].

The mechanical properties of the SMFS base material AlCu5 have been considered using the bilinear material model with von Mises plasticity: Young’s modulus *E* = 70 GPa, Poisson’s ration ν = 0.35, yield stress σ*_y_* = 200 MPa, tangent modulus *E*_t_ = 200 MPa and failure strain ε_f_ = 0.5. When the plastic strain in a finite element exceeds the prescribed failure strain, the finite element is effectively removed from the finite element model.

**Figure 27:**
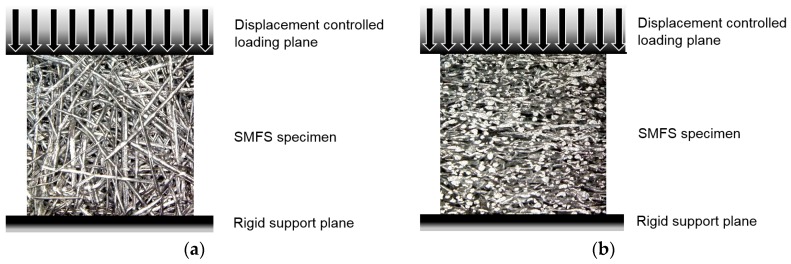
Applied boundary conditions for displacement controlled loading of SMFS specimens: (**a**) loading direction parallel to main fiber orientation; (**b**) loading direction perpendicular to main fiber orientation

The boundary conditions of the finite element analysis combine displacement controlled loading, a fixed planar rigid wall at the opposite specimen’s surface simulating uniaxial compressive loading and the double symmetry boundary conditions on the two adjacent vertical surfaces (Figure 27). Two dynamic loading conditions have been considered, achieving the strain rates 30/s (equal to the experimental testing) and 100/s. In order to computationally study also the influence of the anisotropy of the SMFS two loading directions have been considered in the study: (i) loading parallel to the main fiber orientation; and (ii) loading perpendicular to the main fiber orientation. All nodes of the models were included in an automatic single surface contact accounting for friction with the coefficient of 0.5. The computational model has been analyzed using the commercial engineering software LS-DYNA MPP R7 (single precision) based on the explicit integration scheme [24]. Computational analyses have been performed using the HP ProLiant DL380p cluster with Intel Xeon E5-2670 processors (HP Inc., Palo Alto, CA, USA) and 128 GB of RAM per processor. From 32 to 64 processor cores have been used per simulation, resulting in computational times from 100 up to 400 (for lower velocity loading case) wall-clock hours. From the forces recorded at the rigid support plane, the displacements of the loading plane and the initial dimensions of the SMFS models, the compressive stress-strain data have been evaluated.

## 5. Conclusions

This study provides experimental and numerical results for sintered aluminum fiber structures regarding their energy absorption and the influence of structural anisotropy both under quasi-static and dynamic loading. In addition the well-known influence of the relative density on the mechanical properties, it was found that the direction of loading with regard to the anisotropy of the fiber structure also exerts a significant influence. Moreover, the global deformation and failure mechanisms are visibly different. Numerical simulation based on CT scans confirmed a high anisotropy of sintered metal fiber structure (SMFS) specimens. In the case of the loading direction parallel to the main fiber orientation they revealed that the deformation is distributed along the metallic fibers, while in the case of loading perpendicular to the main fiber orientation, a layer-wise deformation mode has been observed.

The observed stress-strain curves follow the typical pattern of open- and closed-cell cellular metals under compressive loading: an initial strong rise in stress (quasi-elastic region) is followed by a comparably moderate rise in stress (plateau region; characterized mainly by bending and occasional failure of the struts or cell walls) and a third region with another strong rise in stress (densification region; formerly separate struts or cell walls start touching each other, heavy deformation and failure of single struts or cell walls occurs). In particular, loading parallel to the main fiber orientation results in a more “typical” compression behavior: a pronounced quasi-elastic rise in the beginning, a comparably long and flat plateau region, and finally a strong rise in stress due to the onset of densification. This observation is independent of the speed of deformation examined in the reported experiments.

The energy absorption during dynamic loading was found to be smaller than under quasi-static loading. This was attributed to dynamic softening resulting from a rise in temperature of the samples during testing. Due to the pronounced orthotropy of the fiber structures, a strong dependence of the deformation behavior on the direction of loading can be observed. *i.e.*, loading parallel to the preferred fiber orientation results in a strong expansion of the samples accompanied by an initially- strong rise in stress, whereas loading perpendicular to the main fiber orientation leads to almost no changes in the horizontal projection of the sample shape.

The performance of the aluminum fiber structures in terms of energy absorption was compared to that of a commercially available closed-cell high-density polymer foam. At comparable stress and strain levels, the weight specific performance of an aluminum fiber sample with a relative density of 0.16 reached 12% and the volume specific performance 50% of the values of the polymer foam. This is better than expected and could be further optimized by using materials with better strength-to-weight ratios, such as high-strength aluminum alloys, as well as titanium or steel fibers.

A shortcoming of this study is the use of different parent structures and sample geometries for the sample sets for quasi-static and dynamic testing. Although it is most likely that the relative density of the fiber structure and the direction of loading constitute the most relevant parameters for the mechanical performance, further studies should be carried out on sample sets prepared from the same parent structure. Additionally, future work should also provide more insight into the microstructure before and after testing in order to provide a better understanding of the governing deformation and failure mechanisms on the microscopic level.

## Figures and Tables

**Table 1:** Properties of the fibers that have been used for sample-making.

	Quasi-Static Test Samples	Dynamic Test Samples - Mean Relative Density 0.3	Dynamic Test Samples - Mean Relative Density 0.16
Composition	AlCu5	AlCu5	AlCu5
Bulk Density (g/cm^3^)	3.0	3.0	3.0
Mean Fiber Length (mm)	7.6	9.6	8.6
Mean Equivalent Fiber Diameter ^1^ (µm)	125	170	110

^1^ Matching diameter of a fiber with circular cross section and identical length and cross-sectional area. The fibers usually show a diameter distribution with a geometrical standard deviation of about 1.5 to 2.

**Table 2:** Number of specimens and compressive dynamic testing parameters.

Number of Specimens	Average Sample Weight (g)	Average Relative Density	Direction of Loading with Respect to Main Fiber Orientation
4	0.92	0.30	perpendicular
4	0.91	0.30	parallel
4	0.46	0.16	perpendicular
4	0.46	0.16	parallel
